# FCDNet: An Efficient and Cost-Effective Strawberry Disease Detection Model for Smart Farming Management

**DOI:** 10.3390/plants15091341

**Published:** 2026-04-28

**Authors:** Ruoyu Ouyang, Junying Jiang, Yujia Shao, Jialei Zhan, Xiaoyu Zhang

**Affiliations:** 1 Department of Computer Science and Engineering, The Hong Kong Polytechnic University, Hong Kong 999077, China; ruoyuouyang@163.com; 2 School of Business, Wuxi Taihu University, Wuxi 214122, China; 001276@wxu.edu.cn (J.J.); shaoyj1@wxu.edu.cn (Y.S.); 3 College of System Engineering, National University of Defense Technology, Changsha 410073, China; xyzhangphd@outlook.com

**Keywords:** strawberry disease detection, object detection, precision agriculture, feature fusion, smart farming management

## Abstract

With the rapid development of precision agriculture and smart farming management, accurate crop disease detection has become a critical tool for optimizing agricultural resource allocation, controlling operational costs, and supporting scientific plant protection strategies. However, real-world field environments are often characterized by strong background interference, multiple concurrent diseases, and fine-grained lesion differences, posing significant challenges to existing detection methods in practical agricultural Internet of Things (IoT) applications. In this paper, we propose Freq-spatial Context Dynamic Network(FCDNet), an efficient and cost-effective detection model tailored for multi-category strawberry disease recognition in complex field management scenarios. The proposed model integrates a Freq-Spatial Feature Module (FSFM), a Context Guide Fusion Module (CGFM), and a Task Align Dynamic Detection Head (TADDH), enabling enhanced expression of high-frequency micro-lesions, adaptive filtering of field background noise, and spatial alignment of classification and regression tasks, while maintaining a lightweight architecture suitable for low-cost agricultural edge devices. Extensive experiments conducted on the newly constructed Strawberry Disease Dataset-7(S7DD) demonstrate that FCDNet consistently outperforms existing mainstream methods, achieving an F1-score of 91.0% and an mAP@0.5 of 94.6%. The model’s architectural robustness and capacity for generalization are further substantiated by evaluations across diverse agricultural datasets using PlantDoc and ALDOD. Ultimately, FCDNet became a practical and cost-effective tool for real-time detection of strawberry diseases, directly supporting more accurate yield forecasting and risk management in smart agriculture systems.

## 1. Introduction

Driven by the rapid advancement of smart agriculture and precision farming, the early automated detection of crop diseases has emerged as a crucial technological approach for safeguarding agricultural economic returns, optimizing resource allocation, and facilitating production decision-making. For high-value crops such as strawberries, rigorous disease monitoring is essential for developing scientific plant protection strategies, including targeted pesticide application and accurate yield prediction. Automated disease perception systems allow agricultural managers to fully evaluate the health of field crops. This gives them objective data to use when assessing the risks of running a farm and developing sustainable agriculture. In real life, though, field environments usually have a lot of background noise, diseases affecting multiple parts of a plant at the same time, and small changes in the shape of disease lesions. Consequently, intelligent disease detection continues to face significant challenges in practical agricultural Internet of Things (IoT) applications.

**Figure 1:**
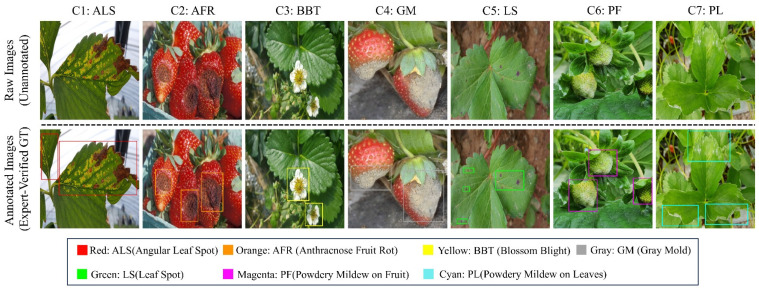
Representative disease samples and annotation-quality visualization of the S7DD. The upper row shows raw images from the seven disease categories, while the lower row presents the corresponding expert-verified ground-truth annotations.

Traditional crop disease monitoring heavily relies on manual scouting. This conventional approach is not only labor-intensive and inefficient but also highly subjective, which frequently leads to delayed management decisions and pesticide overuse, consequently exacerbating the environmental and economic costs of agricultural production [1]. In recent years, non-destructive detection methods based on computer vision have progressively emerged as the mainstream paradigm in agricultural informatization. There are two main types of visual recognition strategies right now: traditional image processing and deep learning-based pattern recognition [2]. Standard methods depend a lot on features that are designed by hand. Because of this, they have trouble working well in different kinds of light and with busy backgrounds, which are common in large-scale precision agriculture. Deep learning methods, on the other hand, solve these problems by giving strong feature representations and making it easier to deploy automatically. Both of these are very important for smart farming systems that keep an eye on things in real time. But even the most advanced models may have trouble keeping both accuracy and robustness in complex situations, especially when the backgrounds are complicated, the pathological variations are subtle, or the lesion size is very different. Figure 1 shows that there are many different kinds of strawberry diseases and that they can look very different from each other. Some examples are spots on leaves, mold on fruit, and blight on flowers. It’s even harder to find the affected areas because the lines between healthy and affected tissues are often not clear. This is because these areas are very sensitive to changes in light and background noise.

In the agricultural applications of object detection algorithms, although two-stage detection methods like Faster R-CNN exhibit outstanding accuracy, their high computational complexity and expensive hardware deployment costs limit their widespread adoption on cost-sensitive agricultural edge computing devices, falling short of the return on investment (ROI) expectations for agricultural IoT construction [3]. In contrast, single-stage detection algorithms, such as You Only Look Once(YOLO), have become the mainstream solutions for real-time intelligent perception in modern agriculture, owing to their efficient inference speed and exceptional cost-effectiveness [4]. However, these methods still exhibit apparent limitations when dealing with the highly variable morphologies of strawberry diseases and the complex, high-frequency environmental noise typical of farmlands [5]. Cutting-edge Transformer-based models (e.g., DETR) excel in global feature modeling, yet their high computational demands make it difficult to directly satisfy the lightweight requirements for real-time field decision-making [6].

While automated strawberry disease detection is essential for precision agriculture, current research still faces several key challenges:

(1) Early-stage strawberry disease lesions are predominantly characterized by fine-grained features and extremely small target scales, making them highly susceptible to interference from farmland backgrounds such as soil and weeds. Existing models lack the capability to extract high-frequency microscopic pathological details, which easily leads to disease misjudgment and subsequent errors in plant protection management decisions (e.g., incorrect pesticide application or delayed treatment).

(2) Single-scale or simplistic feature fusion methods struggle to effectively filter out redundant noise in complex field backgrounds. This results in poor model robustness in multi-scale disease perception, failing to provide high-fidelity data support for comprehensive field health assessments.

(3) When morphological conditions are complex and the boundaries between healthy and diseased tissues blur, standard detection heads frequently experience misalignment between their classification and localization tasks. This disconnect makes it hard to get accurate, plant-level assessments of how bad a disease is, which makes it hard to reliably predict yields and control quality.

This study presents an efficient multi-category strawberry disease detection model, FCDNet, specifically optimized for real-world field conditions to address these challenges. FCDNet focuses on a lightweight architecture and low deployment costs. It has three main modules that work together to improve diagnostic performance in difficult environments. This approach ultimately delivers a highly reliable, cost-effective technological foundation for smart agricultural management. First, a Freq-Spatial Feature Module (FSFM) is proposed, which combines spatial edge operators with a frequency-domain perception mechanism to enhance the feature representation capabilities for minute lesions and high-frequency textures. Second, a Context Guide Fusion Module (CGFM) is made to use context priors to control multi-scale feature fusion and effectively block out background noise in the field. Finally, a Task Align Dynamic Detection Head (TADDH) is introduced. It uses dynamic deformation and feature interaction mechanisms to align classification and regression tasks in space. This greatly improves the accuracy of localization for irregular diseases.

The main contributions of this paper can be summarized as follows:

(1) We construct a high-quality Strawberry 7-class Disease Dataset (S7DD), providing a crucial data benchmark for empirical research in smart agriculture and the development of disease management decision systems in related fields.

(2) We propose the FSFM module, which effectively enhances the model’s perception capability for early-stage minute diseases and complex high-frequency details through spatial-frequency dual-domain collaborative modeling, providing a technical guarantee for the early warning management of farm diseases.

(3) We design the CGFM and TADDH modules, which achieve denoised feature fusion under complex field backgrounds and significantly resolve the task misalignment problem in disease detection, while maintaining extremely low computational overhead (highly adaptable to low-cost agricultural edge devices).

## 2. Related Work

This section will provide a systematic overview and analysis of existing research from three core dimensions: spatial-frequency feature fusion methods, context-guided feature fusion mechanisms, and task alignment strategies in object detection, thereby establishing a solid theoretical and methodological foundation for the proposed FCDNet model.

### 2.1. Spatial-Frequency Feature Fusion Methods

In object detection and disease recognition tasks within open agricultural environments, high-quality feature extraction is central to surpassing the upper bounds of detection performance. In recent years, deep convolutional neural Networks(CNNs) have achieved remarkable progress in the field of plant disease detection owing to their robust spatial feature aggregation capabilities [7]. However, pure spatial domain feature extraction mechanisms have gradually exposed inherent limitations when confronting complex, real-world strawberry disease scenarios. Specifically, spatial convolution relies primarily on local sliding windows; expanding the receptive field typically necessitates the continuous stacking of convolutional layers or the application of downsampling operations. This approach not only easily introduces redundant noise from farmland backgrounds (e.g., soil and weeds), but more critically, the feature smoothing effect inherent in deep networks inevitably leads to a severe loss of microscopic textures and high-frequency edge information of disease lesions, making it difficult to address morphologically variable and minute early-stage diseases [8].

To break through the computational bottlenecks of spatial locality, researchers have shifted their focus to frequency domain analysis, ushering in a new paradigm of feature representation within the spectral domain. Analyzing images from a frequency-domain perspective, low-frequency components typically map to smooth background distributions and global illumination, whereas high-frequency components highly condense discriminative clues such as target edges, contours, and abrupt pathological texture variations. Recent high-level studies demonstrate that integrating the Fast Fourier Transform (FFT) or Discrete Cosine Transform (DCT) into deep networks can capture a global receptive field at an extremely low computational cost. For instance, the Global Filter Network (GFNet) proposed by Rao et al. proved that feature learning in the frequency domain not only effectively avoids information loss caused by spatial downsampling but also significantly enhances the model’s ability to capture high-frequency semantic details [9]. In agricultural vision tasks, the introduction of frequency-domain features (e.g., DCT) has also been proven effective in extracting high-frequency microscopic details that are imperceptible to conventional convolutions, augmenting the model’s discriminative capacity for complex pathological features and thereby mitigating the negative impacts of natural environmental interference [10].

As research deepens, the academic community has gradually recognized that while solely relying on frequency-domain analysis excels at capturing global structures and textures, it is prone to losing precise spatial location information of the targets. Consequently, spatial-frequency feature fusion represents the most cutting-edge trend in the current feature extraction domain. Advanced visual architectures have begun to adopt dual-branch or multi-domain collaborative strategies, attempting to leverage frequency-domain information to reinforce high-frequency details while maintaining spatial localization precision. For example, Qin et al. proposed a spatial-frequency interaction network that achieves complementarity between local semantics and global frequency patterns by establishing feature bridges between the two domains [11]. However, existing dual-domain fusion models (especially frequency-domain variants based on Transformer architectures) are generally accompanied by massive parameter counts and complex cross-domain feature alignment processes, rendering them difficult to directly adapt to the lightweight deployment requirements of agricultural IoT devices.

Meanwhile, to explicitly enhance the network’s sensitivity to boundary features within the spatial domain, classic structured edge operators (e.g., Sobel, Scharr, and Laplacian operators) have experienced a “renaissance” in modern deep learning architectures. Recent studies indicate that embedding non-learnable traditional edge operators into convolutional networks via structural re-parameterization or parallel gradient branches can forcibly guide the network to focus on the physical geometric boundaries of targets, thereby vastly improving the model’s segmentation and detection performance in regions with blurred boundaries between diseased and healthy tissues [12].

In summary, spatial-frequency collaboration and edge-guided strategies have shown great promise in general vision tasks. However, when it comes to strawberry disease detection, there is currently no fusion mechanism that can both extract spatial-frequency details with high precision and be computationally efficient. To fill this research gap, this paper innovatively proposes the FSFM. This module ingeniously combines the explicit edge enhancement capability of the Scharr operator for disease contours in the spatial domain and the dynamic perception capability of the 2D Fast Fourier Transform (2D FFT) for global textures in the frequency domain. It aims to construct a high-fidelity and highly robust spatial-frequency dual-domain feature representation for strawberry disease detection without significantly increasing computational overhead.

### 2.2. Context-Guided Feature Fusion Mechanisms

In object detection tasks, multi-scale feature fusion is crucial for resolving target size variations and fine-grained feature extraction. Traditional feature pyramid networks (FPN) and their variants achieve cross-level information transfer through simple element-wise addition or channel concatenation [13]. However, in agricultural vision, particularly in strawberry disease detection, the extreme scale variation of disease lesions and the surrounding complex environmental noise (e.g., overlapping leaves, soil backgrounds, and water drop reflections) often cause such indiscriminate fusion methods to amplify background noise during feature transfer, thereby obscuring subtle early-stage pathological features.

To address background interference in multi-scale fusion, context-guided and attention mechanisms have gradually become core components of feature extraction architectures. Early foundational research, such as the Squeeze-and-Excitation (SE) network [14] and the Convolutional Block Attention Module (CBAM) [15], achieved adaptive recalibration of feature weights by explicitly modeling interdependencies across channel or spatial dimensions. These mechanisms break the limitations of traditional convolutions that treat all feature maps equally, providing a solid theoretical framework for models to dynamically filter redundant information and focus on key discriminative features.

In recent years, alongside the profound development of smart agriculture, various advanced context-guided mechanisms have been widely introduced into cutting-edge research on plant disease detection in natural environments. For example, addressing the diverse types of strawberry diseases and extremely complex natural backgrounds, recent research by Afzaal et al. deeply integrated an improved attention mechanism with deep convolutional networks, successfully enhancing the model’s feature discriminability for highly confusable disease regions such as strawberry angular leaf spot and Botrytis cinerea [16]. Recent advancements targeting small, multi-scale disease lesions in complex agricultural environments have introduced the enhanced median attention module (MECS), a mechanism that integrates both spatial and channel dimensions. Through global context guidance, this module effectively suppresses background interference caused by natural illumination changes and branch/leaf occlusions, significantly improving the model’s capability to capture fine-grained pathological features [17]. These recent advancements fully demonstrate that dynamically interacting global context prior information with local multi-scale features is the key to enhancing model robustness in real-world farmland environments.

Although existing context-guided and attention mechanisms alleviate background interference to some extent, they still face an intractable challenge when dealing with multi-scale feature fusion: the inherent “semantic gap” between semantic information at different levels. While high-level semantic features contain rich global context, simply fusing them with low-level spatial features easily leads to the “information dilution” phenomenon, resulting in degraded localization accuracy for minute lesions. Meanwhile, overly complex cross-level context interaction modules are often accompanied by massive parameter counts, making them difficult to adapt to the lightweight deployment requirements of agricultural IoT devices.

To break through the aforementioned bottlenecks, this paper proposes an innovative and efficient CGFM. Building upon the SE attention mechanism, this module specifically designs a Context-Guided Cross-Fusion mechanism. With almost no additional computational burden, CGFM can adaptively evaluate and allocate channel weights during the multi-scale feature fusion process. It ingeniously utilizes high-level semantics to precisely guide the extraction of low-level lesion details, while leveraging low-level spatial features to reversely correct the localization deviations of high-level semantics. This architectural design avoids the information loss that happens with traditional fusion methods, resulting in highly discriminative, high-fidelity feature representations that are optimized for detecting multiple types of strawberry disease in messy field conditions.

### 2.3. Task Alignment and Dynamic Detection Heads

In the initial stages of object detection development, single-stage detectors exemplified by traditional YOLO utilized a Coupled Head architecture, concurrently forecasting object categories and bounding boxes via shared feature maps. However, there is a fundamental disparity in feature requirements between classification and localization tasks: classification tends to extract the most discriminative local texture features of the target, whereas localization needs to focus on the target’s global physical geometric boundaries. To resolve this conflict, recent advanced detection frameworks (e.g., YOLOX, YOLOv8) generally introduce a Decoupled Head, processing classification and regression tasks through independent feature branches, which significantly improves the model’s convergence speed and detection performance [18].

Although the decoupled head has achieved tremendous success in general vision tasks, its inherent defects are gradually exposed in complex agricultural vision scenarios. In strawberry disease detection, lesions (such as Botrytis cinerea and Anthracnose) often exhibit irregular morphology, drastic scale variations, and extremely blurred boundaries with healthy tissues. The independent processing mechanism of the decoupled head’s dual branches severs the spatial correlation between classification and localization, making it highly prone to the “Task Misalignment” phenomenon. Specifically, the model might output an extremely high classification confidence for a highly salient local region of the disease, but its predicted bounding box fails to accurately enclose the entire lesion; alternatively, the bounding box might be accurately located, but the classification confidence is low because the feature receptive field deviates from the core pathological region.

To overcome this challenge, the academic community began exploring task alignment mechanisms and dynamic detection head architectures. Task-aligned One-stage Object Detection (TOOD) proposed by Feng et al. not only achieved alignment at the label assignment level but also pioneered a task-interactive feature learning mechanism, laying the foundation for resolving the aforementioned spatial misalignment problem [19]. With the penetration of this concept into the field of smart agriculture, the Dynamic Head has gradually become a cutting-edge approach to enhancing the detection accuracy of complex crop diseases. Recent research shows that dynamic architectures can help with similar problems in agriculture. A 2025 study on corn leaf diseases integrated scale, spatial, and task awareness via a dynamic detection head featuring multiple attention mechanisms, significantly enhancing boundary localization amidst complex natural backgrounds [20]. In a comparable effort targeting multi-scale apple diseases, the implementation of a Dynamic Group Head leveraged dynamic mechanisms to increase spatial fidelity and better align classification and regression tasks, all without sacrificing deployment efficiency [21].

A comprehensive analysis of the aforementioned cutting-edge advancements indicates that achieving deep interaction and alignment between classification and regression tasks through dynamic feature aggregation is the key pathway to breaking through the bottleneck of high-precision detection for morphologically complex diseases. Inspired by this, this paper abandons the conventional static decoupled structure and innovatively proposes a TADDH. The TADDH module introduces lightweight shared convolutions and Group Normalization (GroupNorm) to stabilize training. By leveraging the dynamic deformation capability of Deformable Convolution (DCNv2) and a soft attention feature selection mechanism, it achieves adaptive, perfect alignment of classification and regression features in the spatial dimension. This design not only fundamentally resolves the task misalignment problem in disease detection but also endows the model with the capability for high-precision, flexible localization of irregular strawberry lesion boundaries.

## 3. Method

This section will provide a detailed elaboration of the overall architecture of FCDNet and its core modules.

### 3.1. The Structure of FCDNet

This paper uses YOLOv12n as the baseline model and makes new changes to it to improve its accuracy in detecting strawberry diseases. These changes help with things like lesions that are different sizes, diseases that look the same but are actually different, and backgrounds that make it hard to see the diseases. While newer detection architectures exist, YOLOv12n demonstrates exceptional object detection performance. This is mostly because it strikes a great balance between stability, real-time capability, computational complexity, and performance. This makes it very easy to deploy on devices with limited resources.

**Figure 2:**
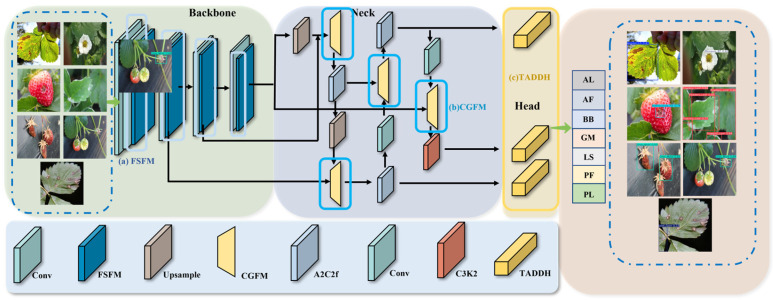
The overall architecture of FCDNet. It consists of three main components: the backbone for feature extraction (integrating the proposed FSFM), the neck for multi-scale feature fusion (integrating the CGFM), and the head for bounding box prediction (integrating the TADDH). Different colored blocks represent specific convolutional or functional operations as denoted in the legend.

Based on these basic improvements, we suggest FCDNet to solve the specific problems that come with finding strawberry diseases. Figure 2 shows that the model is made up of three main parts: a backbone for initial feature extraction, a neck for multi-scale feature fusion, and a final detection head. These parts are all connected in a way that makes it possible to accurately and reliably identify diseases. The backbone is made up of standard convolutional (Conv) layers and our new FSFM module. It is responsible for making the important feature maps that will be used later.

After the features are extracted, the neck combines multi-level features from the back-bone by using cross-layer connections and upsampling to make multi-scale maps for lesions of different sizes. It combines the Upsample, A2C2f, Conv modules, and the new CGFM. Then, it sends the improved features to a detection head with three branches that are specific to each scale for classification and bounding box prediction.

FCDNet makes three big improvements: FSFM at the backbone combines spatial and frequency features to find subtle boundaries; CGFM in the neck uses context-guided attention to highlight important cues and block noise; and TADDH replaces the standard head, aligning classification and localization through dynamic feature selection.

In difficult strawberry farming conditions, these modules work together to make feature representation and detection more reliable. FSFM pulls out structural details even when the lighting is not even or something is blocking the view. CGFM automatically directs focus toward pathological areas, and TADDH combines these features in real time to get precise localization and classification signals for small lesions.

### 3.2. Freq-Spatial Feature Module

Traditional convolutional neural networks primarily perform feature extraction in the spatial domain. Constrained by the inherent limitations of local receptive fields, they often struggle to simultaneously balance fine-grained edge structures and global context information when processing disease images characterized by blurred diseased-healthy boundaries and complex background textures. Specifically, a single spatial convolution is prone to losing high-frequency details when capturing minute texture variations of strawberry diseases; conversely, expanding the receptive field to acquire global information easily introduces irrelevant background noise, thereby limiting the model’s detection performance and generalization capability. Therefore, to address the aforementioned issues, this paper proposes an improved FSFM. This module’s goal is to combine features from both the spatial and frequency domains to better capture spatial structures and frequency patterns at different levels. This will greatly improve the model’s ability to represent features in disease images.

**Figure 3:**
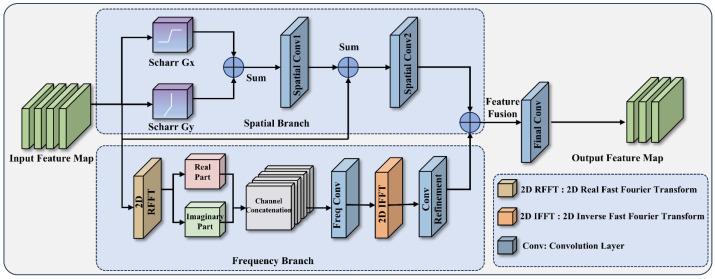
Structural diagram of the FSFM. The module comprises a spatial branch and a frequency branch. In the frequency branch, after transforming the input into the frequency domain via 2D RFFT, the complex output is explicitly decomposed into a Real Part and an Imaginary Part. Following Channel Concatenation, the features are processed by a frequency convolution (Freq Conv) to capture global high-frequency patterns. They are then transformed back via 2D IFFT and passed through a Conv Refinement layer before the final adaptive feature fusion.

To achieve efficient dual-domain feature extraction, we comprehensively consider the complementary advantages of edge operators and the Fourier transform in image analysis. Although standard large-kernel or dilated convolutions can enlarge the receptive field, they usually introduce additional computational burden and remain insufficiently sensitive to the linear edges and abrupt high-frequency variations of disease regions. The core design of FSFM is to integrate structured spatial edge extraction with global frequency-aware representation learning. As illustrated in Figure 3, FSFM adopts a dual-branch parallel architecture composed of a spatial branch and a frequency branch, and the enhanced features from the two branches are finally fused to generate the output representation.

In the spatial branch, the module first processes the input feature map X using a Scharr operator with fixed weights to extract spatial gradient information that is sensitive to disease edges. The Scharr operator computes the gradient magnitudes in the horizontal ($G_{x}$) and vertical ($G_{y}$) directions, which are then weighted and combined to produce the edge-enhanced feature $F_{\mathrm{edge}}$, as expressed in Equation (1):

(1) $$F_{\mathrm{edge}}=0.5\cdot G_{x}\left(X\right)+0.5\cdot G_{y}\left(X\right)$$

Subsequently, to further extract deep spatial semantics and mitigate model overfitting, this branch applies a first spatial convolution (Conv1) to $F_{\mathrm{edge}}$, performs a residual connection (skip connection) with the original input feature map X, and then applies a second spatial convolution (Conv2) to obtain the final spatial feature representation $F_{\mathrm{spatial}}$. This process is formalized in Equation (2):

(2) $$F_{\mathrm{spatial}}={\mathrm{Conv}}_{2}\left({\mathrm{Conv}}_{1}\left(F_{\mathrm{edge}}\right)+X\right)$$

This architecture not only preserves the original contextual information but also effectively emphasizes high-frequency cues, such as the edges of disease regions. Meanwhile, the frequency branch applies a two-dimensional real Fast Fourier Transform (2D RFFT) to project the input feature map X into the frequency domain, where both low-frequency background information and high-frequency lesion details can be captured. As illustrated in Equation (3), the resulting complex-valued spectral representation F(X) is explicitly decomposed into real and imaginary components, which are then concatenated along the channel dimension to form the frequency-domain feature map $F_{\mathrm{freq}\_\mathrm{map}}$.

(3) $$F_{\mathrm{freq}\_\mathrm{map}}=\mathrm{Concat}\left(\mathrm{Real}\left(F\left(X\right)\right),\;\mathrm{Imag}\left(F\left(X\right)\right)\right)$$

After channel concatenation, the frequency-domain features are processed by a frequency convolution layer (Freq Conv) to learn discriminative spectral patterns. The refined spectral representation is then transformed back into the spatial domain through 2D IFFT, followed by a convolutional refinement operation to obtain the final frequency feature representation $F_{\mathrm{freq}}$, as formulated in Equation (4):

(4) $$F_{\mathrm{freq}}={\mathrm{Conv}}_{\mathrm{freq}2}\left(F^{-1}\left({\mathrm{Conv}}_{\mathrm{freq}1}\left(F_{\mathrm{freq}\_\mathrm{map}}\right)\right)\right)$$

After extracting multi-perspective frequency features, the module fuses information via element-wise addition. Finally, the fused feature map is passed through a 1×1 standard convolution layer (Final Conv) to facilitate cross-channel information interaction and dimensional integration. The computation of the final output feature is given in Equation (5):

(5) $$Output=Conv_{1\times 1}\left(F_{spatial}+F_{freq}\right)$$

In summary, the FSFM module effectively overcomes the limitations of traditional convolutional networks by combining the local edge sensitivity of the Scharr operator with the global frequency awareness of the FFT. In this study, we embed this module into the CSP (Cross Stage Partial) framework, constructing a CSP-FreqSpatial module to replace the original C2f structure. This improvement not only introduces additional forward computation paths but also greatly enhances the model’s robustness in recognizing fine-grained details under complex backgrounds [22].

### 3.3. Context Guide Fusion Module

While FPNs and their variants are standard tools for multi-scale feature fusion in object detection, conventional integration methods—such as simple channel concatenation or element-wise addition—often struggle to process representations uniformly across all semantic levels. Consequently, these approaches lack the capacity to selectively amplify relevant contextual information. In the specific context of strawberry disease detection, isolating lesions from healthy foliage remains difficult, requiring a careful balance between fine-grained precision and class differentiation. Without dedicated mechanisms to suppress background interference and prioritize critical features, the propagation of multi-scale information frequently results in semantic confusion. To address this issue, we propose an innovative CGFM, which employs a SE attention mechanism combined with a cross-guidance strategy to adaptively adjust the contextual information weights during the multi-scale feature fusion process.

**Figure 4:**
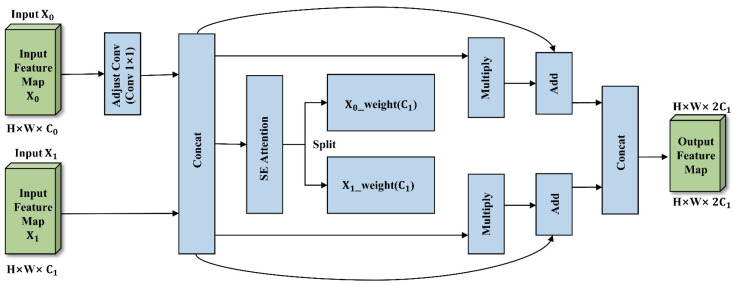
Structural diagram of the CGFM. The module receives multi-scale feature inputs and utilizes an SE attention block to dynamically evaluate channel-wise importance weights. The context-guided cross-fusion mechanism then adaptively integrates these features via cross-addition operations to suppress background noise.

The network architecture of the CGFM is illustrated in Figure 4. This module receives two input feature maps from different levels or scales, $X_{0}\in R^{H\times W\times C_{0}}$ and $X_{1}\in R^{H\times W\times C_{1}}$. To ensure that the final features can be precisely aligned and fused along the channel dimension, the module first adjusts the channel dimension of $X_{0}$. When $C_{0}\neq C_{1}$, a 1×1 convolution (Adjust Conv) is applied to $X_{0}$ to match the channel dimension with $X_{1}$. This preprocessing step is formulated as follows:

(6) $$X_{0}^{\prime }=\left\{\begin{array}{cc} {\mathrm{Conv}}_{1\times 1}\left(X_{0}\right), & \mathrm{if}\;C_{0}\neq C_{1}\\ X_{0}, & \mathrm{otherwise} \end{array}\right.$$

After aligning the dimensions, $X_{0}^{\prime }$ and $X_{1}$ are first concatenated along the channel dimension to form a compact feature map containing rich multi-scale contextual information, denoted as $X_{\mathrm{concat}}$, as expressed in Equation (7):

(7) $$X_{\mathrm{concat}}=\mathrm{Concat}\left(\left[X_{0}^{\prime },X_{1}\right],\;\dim=1\right)$$

The fused feature $X_{\mathrm{concat}}$ is then fed into an SE attention block to compute adaptive channel-wise importance weights. Specifically, a squeeze operation is first performed by global average pooling (GAP) to aggregate the spatial information of $X_{\mathrm{concat}}$ into a channel descriptor z, as formulated in Equation (8):

(8) $$z=GAP\left(X_{\mathrm{concat}}\right)$$

Subsequently, an excitation operation is applied to explicitly model channel interdependencies. The channel descriptor z is passed through a two-layer multilayer perceptron, including a dimensionality-reduction layer with weights $W_{r}$, a ReLU activation function δ(⋅), a dimensionality-expansion layer with weights $W_{u}$, and a Sigmoid activation function σ(⋅), to generate the channel attention vector s, as expressed in Equation (9):

(9) $$s=\sigma \left(W_{u}\;\delta \left(W_{r}z\right)\right)$$

Finally, the unified attention vector s is split along the channel dimension into two channel-weight maps, $W_{0}$ and $W_{1}$, corresponding to the features of the two original branches, as given in Equation (10):

(10) $$W_{0},W_{1}=Split(s,dim=1)$$

The core design of this module lies in its unique Context-Guided Cross-Fusion mechanism. After obtaining the channel-wise attention weights $W_{0}$ and $W_{1}$, each original feature map is first multiplied element-wise with its corresponding weight map to extract the enhanced core features at each level. Instead of a simple output scaling, the module performs a “cross-addition” operation: the enhanced features derived from $X_{1}$ are added to the original features of $X_{0}^{\prime }$, while the enhanced features from the $X_{0}^{\prime }$ branch are incorporated into $X_{1}$’s original features. Mathematically, this is expressed as follows:

(11) $$\begin{array}{c} F_{0}=X_{0}^{\prime }+(X_{1}⊙W_{1})\\ F_{1}=X_{1}+(X_{0}^{\prime }⊙W_{0}) \end{array}$$

Finally, the cross-guided fused features $F_{0}$ and $F_{1}$ are concatenated along the channel dimension to produce the module’s final output:

(12) $$Output=Concat\left(\left[F_{0},F_{1}\right],dim=1\right)$$

Through this simple yet highly efficient cross-reorganization operation, CGFM not only effectively leverages high-level semantic guidance for low-level details but also allows low-level spatial information to refine high-level semantics. This design avoids the information dilution commonly seen in traditional FPNs and significantly enhances the discriminative capability of feature maps for multi-scale strawberry disease detection with minimal additional computational cost.

### 3.4. Task Align Dynamic Detection Head

In existing object detection models, such as standard YOLO architectures, the detection head typically adopts a decoupled structure, separating classification and localization tasks into independent feature extraction branches. However, this design overlooks the intrinsic spatial correlation between tasks: the classification task aligns with the most discriminative local features of the object, whereas the localization task requires precise delineation of the object’s overall contour. Such a structure can exacerbate “task misalignment,” resulting in suboptimal predictions where the predicted class range may be broad yet insufficiently distinguished from the background at each detection scale. To address this issue, inspired by the core idea of TOOD, we propose a novel TADDH [19]. This module performs alignment not only at the feature allocation stage but also at the detection head output by learning task-interactive features, achieving dynamic alignment between classification and regression tasks.

**Figure 5:**
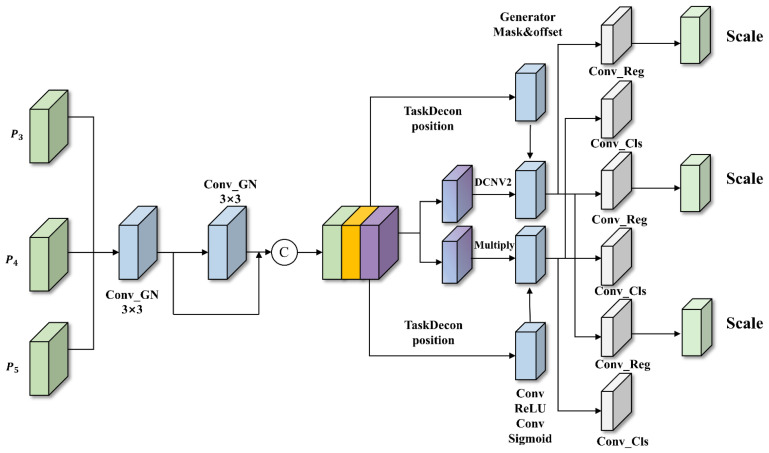
Structural diagram of the TADDH. This dynamic detection head leverages shared convolutions for joint feature extraction, followed by a task decomposition mechanism. It spatially aligns the classification and regression tasks using Deformable Convolution (DCNv2) and a soft attention feature selection mechanism.

The complete network architecture of TADDH is illustrated in Figure 5. For inputs from the multi-scale layers of the object prediction network ($P_{3},P_{4},P_{5}$), TADDH first employs a parameter-sharing mechanism (Shared Convolutions) for feature extraction. The module utilizes consecutive 3 × 3 convolutions combined with Group Normalization (GN) to process the input features, and the outputs of these convolutions are concatenated to form a unified interactive feature $F_{\mathrm{joint}}$. Replacing traditional Batch Normalization with GroupNorm effectively mitigates the instability caused by small batch sizes during training, thereby significantly improving the positional robustness of the detection head [23]. Moreover, the shared convolution design reduces the number of network parameters, rendering the model lightweight and suitable for deployment on resource-constrained devices.

Once the joint feature is obtained, the module applies a task decomposition mechanism combined with global adaptive spatial adjustment to split $F_{\mathrm{joint}}$ into the classification feature $F_{\mathrm{cls}}$ and the regression feature $F_{\mathrm{reg}}$. Subsequently, features are dynamically reshaped to ensure that the regression task adaptively incorporates relevant task-specific information. Specifically, TADDH leverages the fused interactive feature $F_{\mathrm{joint}}$ to generate spatial offsets (Δp) and weight masks (Δm) through convolution kernels and deformable feature extraction. These are then used to process the regression feature $F_{\mathrm{reg}}$ via a deformable convolution network (*DCNv2*), as formulated in Equation (13):

(13) $$F_{\mathrm{reg}\_\mathrm{align}}=\mathrm{DyDCNv}2\left(F_{\mathrm{reg}},\Delta p,\Delta m\right)$$

Through *DCNv2*, the receptive field of the regression features dynamically adapts to the specific morphology and orientation of disease regions, achieving high-precision spatial alignment [24]. Meanwhile, in the classification branch, to suppress interference from complex backgrounds, the module reuses $F_{\mathrm{joint}}$ and applies consecutive convolutions to generate a spatial classification probability map $P_{\mathrm{cls}}$. This probability map is then multiplied element-wise with the stepwise classification feature $F_{\mathrm{cls}}$ to perform dynamic feature selection, as expressed in Equation (14):

(14) $$F_{\mathrm{cls}\_\mathrm{align}}=F_{\mathrm{cls}}⊙P_{\mathrm{cls}}$$

This soft attention mechanism guides the classification branch to focus on the disease core regions that are highly aligned with the bounding box, effectively suppressing misclassification caused by background noise.

Finally, considering the significant differences in object sizes handled by detection heads at $P_{3},P_{4},P_{5}$, TADDH applies a learnable scale layer (Scale Layer) at the output of the regression branch. This layer learns and applies scaling to the features, as shown in Equation (15):

(15) $$Output_{reg}=Scale\left(Conv_{Reg}\left(F_{reg_{a}lign}\right)\right)$$

In short, TADDH gets around the problems with traditional detection heads by combining task decomposition with joint feature extraction. This breaks the barrier between classification and regression. Using DCNv2’s ability to change the shape of space and its flexible feature selection system, TADDH achieves full spatial alignment between classification and regression tasks, which greatly improves the accuracy of strawberry disease detection.

## 4. Experimental Results and Analysis

### 4.1. Dataset Description

To comprehensively and objectively evaluate the performance of the proposed FCDNet model for strawberry disease detection in complex field environments, we constructed a high-quality, multi-class strawberry disease detection dataset, formally named the S7DD. The S7DD contains a total of 2500 high-resolution images. Data acquisition was deliberately carried out under realistic cultivation conditions to capture the types of visual interference commonly encountered in both greenhouse and open-field strawberry production. During collection, special care was taken to account for changes in the environment, such as changing light levels, different background textures, and the fact that leaves and branches often blocked the view. These kinds of things make things much more visually complicated, which is similar to the real-world problems that automated disease detection systems have in real farms.

S7DD takes a more thorough approach to observation by recording pathological symptoms in more than one plant organ, while datasets made from single leaf specimens do not. Disease manifestations are recorded not only on leaves but also on flowers and fruits, allowing the dataset to encompass a wider range of symptom presentations. This multi-organ design makes the dataset more ecologically valid and better shows how diseases develop in natural growing conditions. Within the collected samples, seven diseases commonly observed in major commercial strawberry cultivars are represented. Each disease category exhibits distinctive morphological traits, allowing reliable visual discrimination while also introducing meaningful variability for model training.

Angular Leaf Spot shows the kind of morphological specificity that is in the dataset. In affected leaves, the infection usually causes water-soaked lesions with angular or polygonal outlines. The edges of these lesions are clearly limited by the leaf’s underlying venation structure. This looks very different from Anthracnose Fruit Rot, which mostly shows up on the fruit as dark, sunken depressions in the epidermal tissue. The dataset also includes Blossom Blight, which is when flower tissues die and fall apart over time during the flowering stage. Another common disease in the dataset is Gray Mold, which usually looks like uneven gray-brown fungal growth spreading over infected surfaces. Standard Leaf Spot, on the other hand, looks like well-defined dark circles that are spread out across the leaf blade.

Powdery mildew comes in two forms that look different from each other, which shows that it can infect different parts of plants. When the disease affects fruit, it leaves behind white powdery deposits that make the fruit surface shiny. In foliar infections, on the other hand, the leaves often curl up and have thick colonies of powdery mildew spreading across the tissue.

Domain experts manually annotated all images with bounding boxes to precisely localize the disease regions. Each annotation went through a cross-validation process to make sure that there were no mistakes in the labels. Then, it was exported in the standard YOLO text format (.txt). Figure 1 provides both representative disease samples and annotation-quality visualization for the S7DD. The upper row shows raw images from the seven disease categories, while the lower row presents the corresponding expert-verified ground-truth annotations. To provide a clearer description of the dataset composition, category-wise image-level statistics were compiled for all seven disease categories. The image-level statistics indicate the number of images containing each disease category. In addition, S7DD was divided into the training, validation, and test sets using an image-level stratified split with a ratio of 8:1:1, so as to preserve the class distribution across subsets as much as possible. The detailed category distributions of each subset are presented in Table 1.

### 4.2. Experimental Environment and Parameter Settings

As shown in Table 2, all experiments in this study were conducted on the same computational platform. All experiments were conducted on a workstation equipped with an Intel Core i5-12600KF CPU, 32 GB of RAM, and a 16 GB NVIDIA GeForce RTX 4060 Ti GPU. The software stack relied on Python 3.10.15 and PyTorch 2.0.0, supported by CUDA 12.8 and cuDNN 9.17. The original dataset images were acquired using a Canon EOS 80D digital camera manufactured by Canon Inc. (Tokyo, Japan), with an initial resolution of 1920 × 1080 pixels. To maintain consistent and fair evaluation, all utilized datasets were partitioned into training, validation, and test subsets using a standard 8:1:1 ratio. To ensure a fair comparison, the proposed FCDNet and all baseline models were retrained from scratch under a unified training protocol during the training phase. All models were trained using the same input resolution of 640 × 640, identical data augmentation strategies, and consistent hyperparameter settings, including 200 epochs, a batch size of 64, an initial learning rate of 0.01, and a weight decay of 0.0005. Such a standardized setting ensured experimental consistency across different architectures and yielded stable validation performance while maintaining a reasonable balance between detection accuracy and computational efficiency.

In addition to the workstation-based evaluation described above, an edge deployment experiment was further conducted on a Jetson platform running NVIDIA JetPack 5.1.2 software to assess the practical inference efficiency and energy consumption of the proposed method in low-power agricultural scenarios.

### 4.3. Evaluation Metrics

To fully test how well the proposed FCDNet can find strawberry diseases, a number of tests were done on the newly created S7DD. We used a number of common evaluation metrics to look at the model from different angles during these tests [25]. For example, we looked at how well it worked overall, how accurate it was at detecting things, and how complicated it was.

F1-score: The F1-score is the harmonic mean of Precision and Recall [26]. It gives a balanced measure of false positives and false negatives. It is commonly employed for assessing overall performance in multi-class object detection tasks. A higher F1-score means that the strawberry disease detection system works better overall. The calculations are defined like this:

(16) $$Precision=\frac{TP}{TP+FP}$$

(17) $$Recall=\frac{TP}{TP+FN}$$

(18) $$F1-score=\frac{2\times Precision\times Recall}{Precision+Recall}$$

where TP, FP, TN, and FN represent true positives, false positives, true negatives, and false negatives, respectively.

mean Average Precision (mAP): The mean of the Average Precision (AP) across all classes is used to calculate mAP, which is one of the most common metrics for object detection [27]. This gives a full picture of how well the model detects objects. This metric shows how well the model works for all seven types of strawberry diseases. Higher mAP values mean better detection performance. The calculation is written as:

(19) $$AP=\int_{0}^{1}\mathrm{Precision}(\mathrm{Recall})\;d\left(\mathrm{Recall}\right)$$

(20) $$mAP=\frac{1}{C}\sum_{i=1}^{C}AP_{i}$$

where C denotes the total number of classes. In this study, both mAP@0.5 and mAP@0.5:0.95 were adopted, representing the mean detection performance at a fixed IoU threshold of 0.5 and across IoU thresholds from 0.5 to 0.95 (in steps of 0.05), respectively.

Parameters (Param): Representing the network’s overall size and storage footprint, parameter count serves as a primary metric for evaluating lightweight architectures [28]. Minimizing parameters reduces memory demands and increases deployment flexibility. Throughout this study, parameter totals are reported in millions (M).

Floating Point Operations (FLOPs): This metric assesses computational complexity by quantifying the total operations required for a single forward pass [29]. A lower FLOP count generally translates to higher inference efficiency, which is a prerequisite for executing models on resource-constrained agricultural IoT devices or mobile hardware.

Frames Per Second (*FPS*): *FPS* tells you how quickly the model can make inferences in real life. Real-time processing is necessary because strawberry disease monitoring systems are often used on edge devices or agricultural robots that do not have a lot of computing power. Higher FPS numbers mean faster and more responsive detection. This is how FPS is figured out:

(21) $$FPS=\frac{1}{T_{\mathrm{avg}}}$$

where $T_{\mathrm{avg}}$ represents the average processing time per strawberry image, including image preprocessing, network inference, and post-processing steps such as non-maximum suppression (NMS).

### 4.4. Comparison Experiments and Analysis

To thoroughly assess FCDNet’s effectiveness, we performed benchmark evaluations against several representative object detection methods. As summarized in Table 3, the comparison encompasses traditional general-purpose detectors (Faster R-CNN, SSD), advanced Transformer-based models (RT-DETR), as well as widely used lightweight YOLO variants. It is crucial to note that the performance metrics of these baseline models were not cited directly from their original publications. Instead, to guarantee an absolutely fair and unbiased comparison, all comparative experiments were strictly reproduced locally on the S7DD. Every model was evaluated under the consistent experimental setup and identical training strategy detailed in Section 4.2.

Compared with classical two-stage and single-stage detectors such as Faster R-CNN and SSD, FCDNet demonstrated overwhelming superiority across all core evaluation metrics. These traditional frameworks showed markedly lower detection accuracy on complex strawberry disease images (mAP@0.5 of only 70.2% and 72.5%, respectively), while their parameter counts (82.5 M and 27.1 M) and computational complexity (370.3 G and 63.4 G) were substantially higher than those of our model. Even compared with the Transformer-based RT-DETR, FCDNet maintained a clear lead in detection accuracy (mAP@0.5 improved by 6.5%) while achieving significantly higher computational efficiency, with its parameter count being only one-tenth of RT-DETR, resulting in an outstanding performance-to-efficiency ratio.

Direct comparisons with other lightweight YOLO-family models further confirmed FCDNet’s advantages. Experimental results demonstrate that, in comparison to the advanced baseline YOLOv12n and other cutting-edge variants in the series (e.g., YOLOv8n, YOLOv10n, and YOLOv11n), FCDNet consistently attained significant enhancements across all metrics. FCDNet improved Precision by 2.7%, Recall by 2.9%, and both mAP@0.5 and mAP@[0.5:0.95] by 3.1% when compared to the baseline YOLOv12n. The results show that the FSFM, CGFM, and TADDH modules work as planned. These parts always make things better, even when they are added to the best detectors, by focusing on fine-grained feature extraction and multi-scale variations in strawberry diseases.

Crucially, FCDNet establishes a new performance benchmark on the S7DD. As highlighted in bold in Table 3, FCDNet achieved state-of-the-art results with mAP@0.5 of 94.6% and the more stringent mAP@[0.5:0.95] of 80.2%. Furthermore, achieving an F1-score of 91.0% demonstrates a strong balance between precision and recall, which is essential for reliable disease detection in real-world agricultural settings. This balance is essential for guiding precise pesticide application and preventing unnecessary agricultural resource waste.

Although FCDNet exhibits a slight increase in parameter count (3.2 M) and computational load (8.5 G) compared with the baseline YOLOv12n, it maintains a high inference speed of 102 FPS on the high-performance NVIDIA GeForce RTX 4060 Ti workstation platform. To further validate its practical deployment feasibility in agricultural edge scenarios, we additionally conducted deployment experiments on the Jetson Xavier NX edge device manufactured by NVIDIA Corporation (Santa Clara, CA, USA). The trained YOLOv12n and FCDNet models were exported to TensorRT engines and evaluated under three precision modes, namely FP32, FP16, and INT8, with a batch size of 1 and an input resolution of 640 × 640. For fairness, the same preprocessing and post-processing pipeline was adopted for both models.

During deployment evaluation, the average inference latency was measured after 50 warm-up iterations over 500 test images, and the corresponding FPS was calculated accordingly. Meanwhile, the average power consumption during continuous inference was recorded using the device monitoring tool. As summarized in Table 4, FCDNet achieved 24.1 FPS, 31.9 FPS, and 38.8 FPS under FP32, FP16, and INT8 deployment modes, respectively, with corresponding average latencies of 41.5 ms, 31.3 ms, and 25.8 ms and average power consumption of 11.7 W, 11.1 W, and 10.6 W. In particular, the FP16 deployment mode provided the most favorable balance between accuracy and efficiency, yielding 94.5% mAP@0.5, 80.1% mAP@[0.5:0.95], 31.3 ms average latency, and 11.1 W average power consumption.

Compared with YOLOv12n, FCDNet exhibits slightly lower inference speed on the Jetson platform due to its higher parameter count and computational complexity, yet it consistently maintains superior detection accuracy across all precision modes. For example, under FP16 deployment, FCDNet improves mAP@0.5 from 91.4% to 94.5% and mAP@[0.5:0.95] from 77.0% to 80.1%, while still preserving competitive real-time capability. These results indicate that FCDNet is not only effective under standard workstation-based evaluation but also feasible for deployment in low-power edge scenarios, thereby strengthening its practical value for intelligent agricultural monitoring.

**Figure 6:**
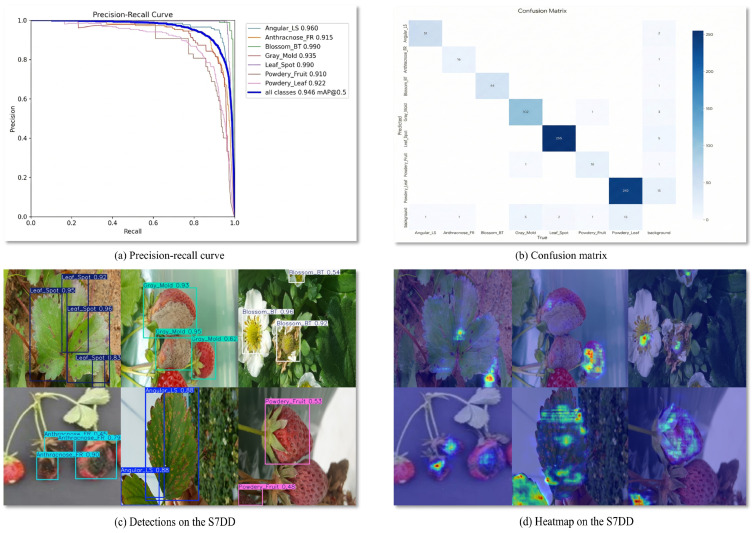
Qualitative analysis results of FCDNet on the S7DD: (**a**) Precision–Recall (P-R) curves for different strawberry disease categories; (**b**) Confusion matrix illustrating the classification performance across all disease classes; (**c**) Representative detection results in real agricultural scenes, with bounding boxes indicating detected diseases on leaves, flowers, and fruits; (**d**) Attention heatmaps highlighting the discriminative pathological regions precisely focused on by the model.

We generated a series of visualizations (Figure 6) to comprehensively evaluate FCDNet’s performance in natural strawberry disease detection scenarios. By examining Precision-Recall curves alongside confusion matrices, bounding box outputs, and attention heatmaps, this figure provides a detailed view of the model’s performance on the S7DD, highlighting both its predictive accuracy and the internal mechanisms by which it emphasizes relevant feature regions.

Figure 6a shows that FCDNet’s P-R curves are mostly in the upper-right area with high scores. This means that it can consistently detect all types of strawberry diseases. Notably, the model achieves near-ideal P-R curves for Blossom Blight and Leaf Spot. The model maintains a high level of accuracy even for classes that are usually hard to see, like Gray Mold and Powdery Mildew on Fruit. This shows that FCDNet successfully lowers the number of missed detections while also lowering the number of false positives. This is in line with the high quantitative performance seen in the mAP@0.5 metric (94.6%).

The confusion matrix for the S7DD is shown in Figure 6b. The high values along the diagonal of the confusion matrix show that FCDNet can tell the difference between all seven types of strawberry diseases very well. This consistency shows that the model can tell the difference between sick parts of the plant’s physiology. Some samples were put in the wrong group because they looked alike or because of reflections in the background. However, these errors mostly happened when the lighting changed or the leaves were blocked, which are common in natural field environments. Still, FCDNet has the best overall classification and localization performance in the industry.

Figure 6c provides examples of FCDNet’s detection results on real field images. The model accurately locates multi-scale disease targets on leaves, flowers, and fruits, producing highly precise bounding boxes and high-confidence class predictions, even when the background is complicated (for example, with soil and weeds) and the lighting changes a lot. FCDNet shows great and stable detection performance even when there are a lot of diseases, like when Leaf Spot infections happen at the same time or when different parts of a plant get infected at the same time. This shows that it could be useful for smart agricultural monitoring.

Figure 6d shows the feature attention heatmaps associated with the detection samples. The visualizations indicate that the highly activated regions are mainly concentrated around pathological characteristics, such as leaf necrosis and fungal lesions, whereas relatively limited responses are observed in most background areas, including healthy foliage and soil. These results qualitatively suggest that FCDNet tends to focus on disease-relevant regions while suppressing part of the irrelevant background interference. However, such heatmap evidence alone is still insufficient to fully verify the robustness of the model under natural and uncontrolled conditions.

**Figure 7:**
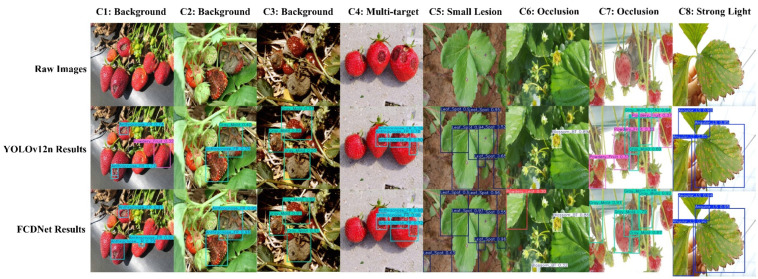
Qualitative comparison of detection results on real images collected from natural and uncontrolled environments. The first row shows the raw images, the second row presents the detection results of YOLOv12n, and the third row shows the corresponding results of FCDNet. The selected examples include cluttered backgrounds, background interference, multi-target scenes, small early-stage lesions, occlusion, and strong illumination conditions.

To further examine this point, we additionally provide a qualitative comparison on real images collected from natural and uncontrolled environments, as shown in Figure 7. The selected examples include cluttered backgrounds, background interference, multi-target scenes, small early-stage lesions, occlusion, and strong illumination conditions. The comparison results show that, relative to the baseline YOLOv12n, FCDNet produces more accurate localization and more stable predictions in these challenging real-world scenarios, especially when the disease regions are small, partially occluded, or embedded in complex backgrounds. These additional results provide more direct evidence of the practical robustness of FCDNet in realistic agricultural environments.

The visual analysis in Figure 6, together with the additional qualitative validation in Figure 7, demonstrates that FCDNet achieves state-of-the-art quantitative results on the S7DD while maintaining strong qualitative performance under both standard test samples and more challenging real-world conditions. Together, these results confirm the effectiveness of the proposed architecture and highlight its practical utility for automated strawberry disease monitoring in complex field environments.

**Figure 8:**
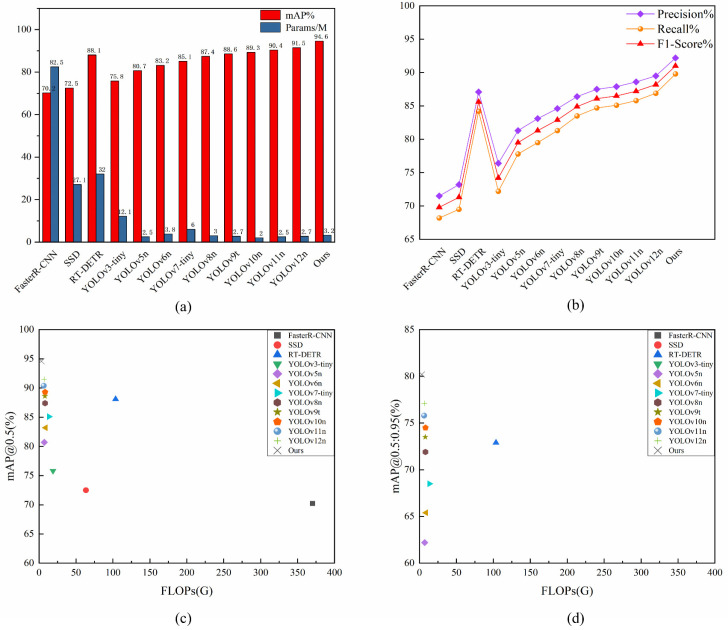
Visual comparison of FCDNet with baseline models under different evaluation metrics. (**a**) Comparison of mAP@0.5 versus the total number of parameters (Params/M); (**b**) Line chart comparing Precision, Recall, and F1-Score across all tested models; (**c**) Scatter plot illustrating the trade-off between computational complexity (FLOPs) and mAP@0.5; (**d**) Scatter plot illustrating the trade-off between computational complexity (FLOPs) and the more stringent mAP@[0.5:0.95] metric.

Figure 8 shows a multi-dimensional visualization analysis that makes it easier to compare FCDNet to other detection methods. Figure 8a shows how mAP@0.5 changes with the total number of parameters. FCDNet has the best detection accuracy at 94.6%, even though it only has 3.2 million parameters to work with. This shows that there is an ideal balance between model size and overall performance.

Figure 8b shows how Precision, Recall, and F1-score compare for all the methods that were tested. FCDNet always got the highest scores. The model shows that it has the perfect balance between precision and recall by getting the highest F1-score. In real-world farming settings, this balance is important for keeping diseases from going undetected while also stopping false positives.

Figure 8c shows the relationship between computational complexity (FLOPs) and mAP@0.5. From the scatter plot, it is clear that FCDNet (black “X” marker) is positioned in the upper-left region of the chart, achieving top-tier detection performance at extremely low computational cost (8.5 GFLOPs). This further confirms the model’s leading trade-off between efficiency and accuracy.

Figure 8d plots computational complexity against the more rigorous mAP@[0.5:0.95] metric. Even under these stricter evaluation criteria, FCDNet remains optimally positioned in the upper-left quadrant of the graph. This demonstrates that the network delivers high localization precision across multiple IoU thresholds, rather than merely succeeding at the baseline standard.

As illustrated in the four subplots, FCDNet outperforms traditional general-purpose detectors like Faster R-CNN and performs competitively against state-of-the-art architectures such as RT-DETR. Furthermore, when evaluated alongside advanced lightweight models like the recent YOLOv12n, our network secures leading results across all primary metrics. Ultimately, this demonstrates that FCDNet achieves an optimal trade-off between detection accuracy and computational efficiency for complex agricultural deployments.

**Figure 9:**
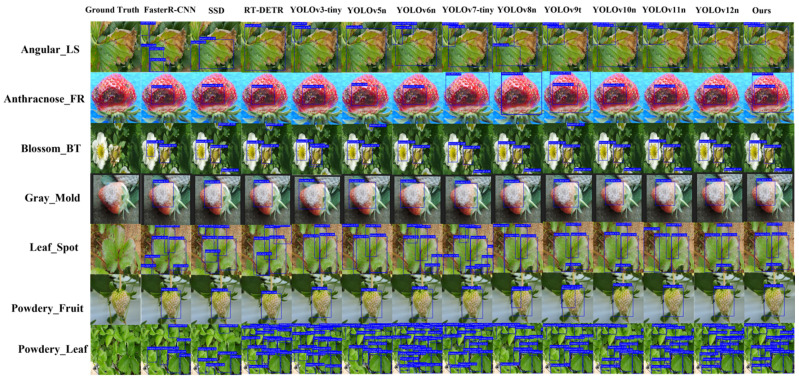
Visualization results obtained from comparison experiments on the S7DD. Each column represents the detection outputs from a specific baseline model, while each row illustrates a distinct strawberry disease category. The bounding boxes indicate the predicted lesion locations and corresponding confidence scores. For detailed sample size distributions and image acquisition parameters (e.g., equipment and resolution), please refer to Section 4.1 (Table 1) and Section 4.2.

To qualitatively evaluate FCDNet’s performance in real-world agricultural detection scenarios, we conducted a visual analysis of seven representative strawberry disease samples from the S7DD. As detailed in Section 4.1 (Table 1) and Section 4.2, the visualization covers various disease categories with diverse sample sizes, initially captured at a resolution of 1920 × 1080 and standardized to 640 × 640 inputs. As shown in Figure 9, the detection results of FCDNet were directly compared with a series of baseline models, ranging from classical frameworks such as Faster R-CNN and SSD to multiple advanced YOLO-family variants, ensuring a comprehensive assessment.

Traditional detectors, like Faster R-CNN and early YOLO variants, often miss detections or make extra bounding boxes over the same areas when there are small, densely distributed, and scale-variable lesions on leaves, like Leaf Spot and Angular Leaf Spot. FCDNet, on the other hand, is very strong and can find and accurately locate even the smallest lesions, many of which are well hidden in the plant tissue. The FSFM module, which combines features from both the spatial and frequency domains, is mostly what makes it easier to see small details. The model accurately depicts disease-specific patterns by integrating high-frequency edges with subtle textural variations.

Gray Mold, Anthracnose Fruit Rot, and Blossom Blight are diseases that make it hard to find the exact location of lesions because they often look like uneven mold layers or shallow depressions that blend in perfectly with healthy tissue. In these situations, baseline models frequently create bounding boxes that are either excessively large or misaligned. The pictures show that FCDNet can correctly draw these strange shapes. The TADDH module makes this level of accuracy possible. By combining a task-alignment mechanism with dynamic deformable convolutions, the network can adapt to the real geometric shapes of the lesions, capturing their edges in a more accurate and detailed way.

Moreover, in detecting Powdery Mildew on Fruit and Powdery Mildew on Leaves, the powdery pathological features are easily confounded with reflective highlights on fruit surfaces or leaf tissues under natural lighting. Other comparative models frequently exhibit localization offsets or even severe misclassifications in such scenarios. FCDNet successfully overcomes these interferences, accurately distinguishing genuine disease textures from environmental reflections and avoiding localization errors. These results objectively validate the critical role of the CGFM module in guiding effective contextual feature integration while suppressing background noise interference.

### 4.5. Comparative Experiments on Different Datasets

We conducted comprehensive evaluations on two public datasets, PlantDoc and ALDOD, to thoroughly assess the architectural adaptability and generalization capability of FCDNet across diverse agricultural scenarios.

The PlantDoc dataset contains 29 disease categories spanning various crops, such as apple, maize, potato, and tomato. Because of this broad species diversity and its complex field backgrounds, PlantDoc presents a substantial domain shift from our primary S7DD data. In contrast, ALDOD provides a high-quality benchmark focused exclusively on apple leaf diseases. Its acquisition conditions, background environments, and image styles differ markedly from S7DD, representing another form of domain shift. Together, these two datasets allow evaluation of the model’s generalization and domain-adaptation capabilities along two dimensions: cross-species diversity and distribution differences of similar diseases.

To objectively assess the architectural generalizability of the proposed methods on different data distributions, a strict evaluation protocol was adopted: both FCDNet and all comparative models were independently trained from scratch and evaluated on the PlantDoc and ALDOD datasets, strictly following their respective standard training and testing splits. Moreover, the preprocessing pipeline used during testing was kept identical to that of S7DD to rigorously evaluate the intrinsic capacity of the model to adapt to out-of-domain features.

Table 5 and Table 6 show how well FCDNet did compared to baseline methods on the two datasets. Even with significant changes in the domain, the model shows strong generalization. As shown in Table 5, FCDNet improves the mAP@0.5 score by 1.1% (from 78.5% to 79.6%) and the mAP@[0.5:0.95] score by 1.4% (from 60.1% to 61.5%) on the PlantDoc dataset. This is compared to the already strong YOLOv12n baseline. The FSFM and CGFM modules work together to extract transferable, high-frequency disease features while blocking out background noise in new environments. This is a big reason why performance can be maintained across species.

As shown in Table 6, FCDNet once again shows a clear performance advantage on the ALDOD dataset. The model consistently beats YOLOv12n (95.2% and 81.3%) and other state-of-the-art methods of the time, with mAP@0.5 and mAP@[0.5:0.95] scores of 96.1% and 82.5%, respectively. These results show that the TADDH module’s dynamic task-alignment mechanism lets the network adapt well to changes in the style and lighting of images across datasets. Consequently, the module can accurately represent the genuine physical contours of the target regions, even when utilized with data from previously unexamined domains.

The results from both datasets show that the FSFM, CGFM, and TADDH modules work well together to help FCDNet deal with the specific problems that the S7DD presents. The network not only meets this main goal, but it also shows strong generalization across domains and practical robustness, which means it would work well in real-world situations where there is variability across domains.

### 4.6. Ablation Study

To validate the effectiveness of FCDNet’s core components and their contributions to overall performance, a series of rigorous ablation experiments were conducted on the S7DD. The results are presented in Table 7. Using the state-of-the-art YOLOv12n as the baseline, we incrementally integrated the Frequency-Spatial Feature Module (FSFM), the Context-Guided Fusion Module (CGFM), and the Task Align Dynamic Detection Head (TADDH) to systematically analyze the specific role of each module in addressing the challenges of strawberry disease detection.

Although the baseline YOLOv12n model provides high inference speed and strong initial performance, its feature extraction capability remains limited when handling complex field backgrounds and multi-scale fine-grained lesions. Experiments that add each module separately show very clearly how useful these targeted improvements are. Adding the FSFM module raised the mAP@0.5 by 1.0% and the F1-score by 0.9%, which shows that spatial-frequency feature fusion is important for resolving complex lesion edges and micro-textural details. The CGFM module also helped reduce background noise during multi-scale fusion, which led to a 1.1% mAP@0.5 improvement with very little extra computing power. The inclusion of the TADDH module further enhanced performance, demonstrating the efficacy of dynamic deformable mechanisms and task alignment in adapting to irregular disease morphologies.

Combination experiments further elucidated the synergistic effects of these feature-enhancement strategies. The mAP@0.5 went up to 93.5% when the FSFM and CGFM modules were combined. This suggests that the FSFM’s multi-scale frequency-spatial representations help the CGFM’s adaptive contextual filtering. The CGFM and TADDH pairing had the best performance of all the two-module setups, with a mAP@0.5 of 93.7% and an F1-score of 90.2%. These results show that adding high-quality, low-noise contextual features to the detection head greatly improves the dynamic alignment of classification and regression tasks.

Adding all three core modules to FCDNet makes it possible to find a good balance between speed and accuracy. Table 7 shows that this fully configured model performs best on the main evaluation metrics, with an F1-score of 91.0%, a mAP@0.5 of 94.6%, and a strict mAP@[0.5:0.95] of 80.2%. The results show that the architecture works well together: the FSFM module gets all the fine-grained pathological details, the CGFM keeps important contextual information while getting rid of background noise, and the TADDH uses these refined representations to make accurate spatial alignments and task-specific predictions. The full configuration does add a moderate amount of parameters (3.2 M) and processing power (8.5 GFLOPs) compared to the very lightweight baseline. However, FCDNet still has a high inference speed of 102 FPS, which makes it suitable for high-precision, real-time intelligent detection in real-world agricultural settings.

**Figure 10:**
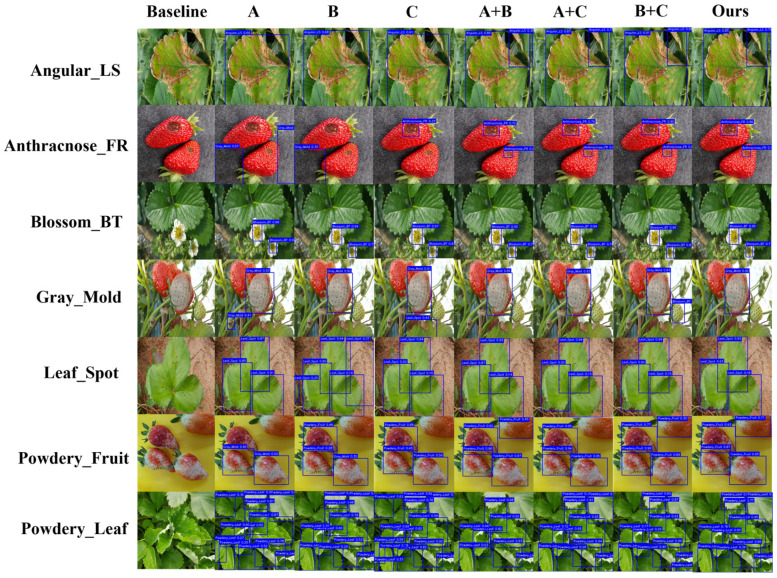
Qualitative ablation comparison of FCDNet under different module configurations, demonstrating the effectiveness of FSFM, CGFM, and TADDH in improving detection accuracy and robustness for strawberry disease detection. (Note: A represents FSFM, B represents CGFM, and C represents TADDH).

To provide a more intuitive analysis of the impact of each module on detection performance, Figure 10 presents visualized ablation results of FCDNet under different module combinations. From the figure, it can be observed that the baseline model still exhibits missed and false detections in certain complex natural field scenarios, particularly when lesions are dense and small (e.g., Leaf Spot), irregular with blurred edges (e.g., Gray Mold), or affected by reflective highlights (e.g., Powdery Mildew on Fruit). In these cases, bounding box localization is unstable, and some targets may even be missed entirely.

When the FSFM module is added by itself (Column A), the model gives more accurate answers to high-frequency lesion edges and local micro-textures. The ability to capture small lesions, like Angular Leaf Spot and Leaf Spot, has gotten a lot better. This shows that the dual-domain spatial-frequency fusion does a good job of improving the modeling of fine-grained pathological features.

A closer look at Column B, which shows the CGFM module’s contribution, shows that it has improved the overall consistency of detection in complex backgrounds. There are a lot fewer false positives now that reflections from the ground or leaf surface do not happen as often. This shows that the context-guided mechanism can get rid of background noise that is not useful and make the model stronger in tough situations.

The model’s focus on irregularly shaped lesions, like Anthracnose Fruit Rot and Blossom Blight, is even stronger with the TADDH module (Column C) added. High-confidence detection regions are more concentrated, and the alignment of bounding boxes with the true physical contours of lesions is significantly improved, greatly reducing localization errors.

Finally, when all three modules are activated simultaneously (Ours column, i.e., A + B + C), FCDNet exhibits the most stable and accurate detection across multi-part strawberry disease scenarios. The model can perfectly capture multi-scale lesions while ensuring detection completeness, maintaining extremely high bounding box localization accuracy and confidence scores.

## 5. Conclusions and Future Work

### 5.1. Summary of the Study

Addressing the challenges of unexpected economic losses and resource waste caused by crop diseases in modern agricultural production, this study proposes FCDNet, a multi-class strawberry disease detection model designed to balance low-cost deployment with high-precision perception from the perspective of refined farm management and lean production. Conventional disease monitoring depends heavily on manual inspection, a process burdened by escalating marginal costs and delayed management responses. Even though computer vision offers an automated option, current algorithms often do not find a good balance between strong performance in difficult areas and a good return on investment. FCDNet directly addresses this problem by bringing together advanced technical skills with the practical and economic needs of farming.

We tried out FCDNet on our new S7DD, and its combination of the FSFM, CGFM, and TADDH modules worked great for finding hard-to-find, multi-scale field lesions. The architecture is very efficient, with a real-time inference speed of 102 frames per second (measured on the workstation GPU) and only 3.2 million parameters. The model scored 94.6% mAP@0.5 and 91.0% F1 on the S7DD. Evaluations across diverse agricultural datasets using PlantDoc and ALDOD demonstrated its capacity for effective architectural generalizability. FCDNet’s best balance of precision and recall lowers the risk of losing yield because of missed detections and keeps the costs of pesticide over-application due to false positives as low as possible.

Beyond functioning as a standalone detection model, FCDNet can be readily integrated into agricultural IoT systems to facilitate real-time data acquisition. Its highly lightweight design allows deployment on low-cost edge devices or autonomous inspection robots with minimal barriers, providing agricultural managers with real-time, high-fidelity field health assessment data.

### 5.2. Limitations and Future Directions

Despite achieving state-of-the-art performance on the S7DD, the current study acknowledges several critical limitations. First, the edge deployment analysis is presently limited to a single Jetson platform and does not yet cover a wider range of low-power devices (e.g., other Jetson series, Raspberry Pi-based accelerators, or heterogeneous edge hardware). Second, the model’s robustness under extreme environmental conditions—such as nighttime capturing scenarios or intense direct sunlight—remains underexplored. Third, accurately disentangling densely co-occurring multiple diseases on a single plant organ presents an ongoing challenge. Finally, like many data-driven models, FCDNet may exhibit sensitivity to annotation noise and varying labeling quality in large-scale agricultural datasets.

To overcome these limitations, our future work will unfold in several compelling directions. Practically, we will prioritize bridging the hardware deployment gap through on-site tests on diverse edge computing platforms to comprehensively evaluate real-time latency and power consumption. Algorithmically, we aim to integrate semi-supervised learning paradigms to reduce reliance on perfectly annotated data and enhance the model’s robustness against annotation noise. We also plan to employ domain adaptation techniques to improve generalization under extreme lighting shifts. Ultimately, by integrating this robust visual perception model with crop economic loss assessment frameworks and supply chain decision systems, we aim to enable plant protection management to fully shift from a traditional “reactive response” to a modernized paradigm of “proactive warning and precise intervention.”

## Figures and Tables

**Table 1:** Image-level distribution of the seven disease categories in the S7DD.

Category ID	Disease Category	Total Images	Train Images	Val Images	Test Images
0	Angular Leaf Spot	430	344	43	43
1	Anthracnose Fruit Rot	100	80	10	10
2	Blossom Blight	210	168	21	21
3	Gray Mold	480	384	48	48
4	Leaf Spot	610	488	61	61
5	Powdery Mildew on Fruit	140	112	14	14
6	Powdery Mildew on Leaves	530	424	53	53

**Table 2:** Experimental environment and parameters.

Designation		Configuration Information
Hardware environment	CPU	Intel(R) Core (TM) i5-12600KF
	RAM	32 GB
	Video memory	16 GB
	GPU	NVIDIA GeForce RTX 4060 Ti
Software environment	Python	3.10.15
	Pytorch	2.0.0
	CUDA	12.8
	cuDNN	9.17
Parameters	Size of input images	640 × 640
	Learning rate	0.01
	Epochs	200
	Batch size	64
	Decay	0.0005

**Table 3:** Comparative experimental results of ours and mainstream models on S7DD.

Model	Precision/%	Recall/%	mAP@0.5/%	mAP@[0.5:0.95]/%	F1-Score/%	Param/M	FLOPs/G	FPS
FasterR-CNN [30]	71.5	68.2	70.2	48.6	69.8	82.5	370.3	56
SSD [31]	73.2	69.5	72.5	50.8	71.3	27.1	63.4	65
RT-DETR [32]	87.1	84.2	88.1	72.9	85.6	32	103.6	24.1
YOLOv3-tiny [33]	76.4	72.2	75.8	54.3	74.2	12.1	18.9	126.2
YOLOv5n [34]	81.3	77.8	80.7	62.2	79.5	2.5	7.1	109.4
YOLOv6n [35]	83.1	79.5	83.2	65.4	81.3	3.8	8.9	100.2
YOLOv7-tiny [36]	84.6	81.3	85.1	68.5	82.9	6.0	13.4	148.3
YOLOv8n [37]	86.4	83.5	87.4	71.9	84.9	3.0	8.1	152.6
YOLOv9t [38]	87.5	84.7	88.6	73.5	86.1	2.0	7.9	124
YOLOv10n [39]	87.9	85.1	89.3	74.5	86.5	2.6	8.2	113.6
YOLOv11n [40]	88.6	85.8	90.4	75.8	87.2	2.5	6.3	143.4
YOLOv12n [41]	89.5	86.9	91.5	77.1	88.2	2.7	6.7	104.1
Ours	92.2	89.8	94.6	80.2	91.0	3.2	8.5	102

**Table 4:** Edge deployment performance of YOLOv12n and FCDNet on the Jetson Xavier NX.

Model	Precision	Latency/ms	Power/W	mAP@0.5/%	mAP@[0.5:0.95]/%	Engine Size/MB	Param/M	FLOPs/G	FPS
Yolov12n	FP32	36.2	10.8	91.5	77.1	11.2	2.7	6.7	27.6
Yolov12n	FP16	28.4	10.2	91.4	77.0	5.9	2.7	6.7	35.2
Yolov12n	INT8	23.1	9.8	90.9	76.4	3.4	2.7	6.7	43.3
FCDNet	FP32	41.5	11.7	94.6	80.2	12.9	3.2	8.5	24.1
FCDNet	FP16	31.3	11.1	94.5	80.1	6.8	3.2	8.5	31.9
FCDNet	INT8	25.8	10.6	93.7	79.0	3.9	3.2	8.5	38.8

**Table 5:** Comparative experiments on the PlantDoc dataset.

Model	Precision/%	Recall/%	mAP@0.5/%	mAP@[0.5:0.95]/%	F1-Score/%	Param/M	FLOPs/G	FPS
FasterR-CNN	53.1	48.2	52.4	31.2	50.5	82.5	370.3	54
SSD	55.5	51.4	55.2	33.5	53.4	27.1	63.4	64
RT-DETR	73.5	69.8	74.0	54.5	71.6	32	103.6	23.8
YOLOv3-tiny	58.2	54.5	58.6	36.8	56.3	12.1	18.9	125.2
YOLOv5n	64.5	60.2	65.4	44.2	62.3	2.5	7.1	108.1
YOLOv6n	67.8	63.5	68.2	47.5	65.6	3.8	8.9	99.3
YOLOv7-tiny	69.5	66.1	70.5	50.2	67.8	6.0	13.4	147.2
YOLOv8n	72.1	68.5	72.8	53.4	70.3	3.0	8.1	151.6
YOLOv9t	74.6	71.2	75.1	55.8	72.9	2.0	7.9	122
YOLOv10n	75.8	72.5	76.2	57.2	74.1	2.6	8.2	111.6
YOLOv11n	76.9	73.8	77.4	58.5	75.3	2.5	6.3	141.4
YOLOv12n	77.5	74.6	78.5	60.1	76.0	2.7	6.7	103.3
Ours	78.2	75.4	79.6	61.5	76.8	3.2	8.5	100

**Table 6:** Comparative experiments on the ALDOD dataset.

Model	Precision/%	Recall/%	mAP@0.5/%	mAP@[0.5:0.95]/%	F1-Score/%	Param/M	FLOPs/G	FPS
FasterR-CNN	75.1	71.2	73.8	52.6	73.1	82.5	370.3	57
SSD	76.8	73.5	76.5	55.2	75.1	27.1	63.4	66
RT-DETR	90.2	87.0	91.8	76.8	88.6	32	103.6	25.1
YOLOv3-tiny	79.5	75.8	79.2	58.5	77.6	12.1	18.9	127.8
YOLOv5n	84.5	80.6	84.5	66.8	82.5	2.5	7.1	111.1
YOLOv6n	86.2	82.4	86.8	69.5	84.3	3.8	8.9	102.3
YOLOv7-tiny	87.6	84.1	88.5	72.4	85.8	6.0	13.4	149.8
YOLOv8n	89.5	86.2	90.6	75.2	87.8	3.0	8.1	154.2
YOLOv9t	90.8	88.2	92.5	77.9	89.5	2.0	7.9	125
YOLOv10n	91.5	89.0	93.2	78.8	90.2	2.6	8.2	116.3
YOLOv11n	92.6	90.1	94.1	80.0	91.3	2.5	6.3	146.2
YOLOv12n	93.5	91.2	95.2	81.3	92.3	2.7	6.7	106.1
Ours	94.2	92.5	96.1	82.5	93.3	3.2	8.5	105

**Table 7:** Ablation study results on S7DD under different module combinations.

Configuration	F1-Score/%	mAP@0.5/%	mAP@[0.5:0.95]/%	Param/M	FLOPs/G	FPS
Baseline (YOLOv12n)	88.2	91.5	77.1	2.7	6.7	104.1
Baseline + FSFM	89.1	92.5	78.0	2.9	7.3	103.3
Baseline + CGFM	89.3	92.6	78.2	2.8	7.1	103.6
Baseline + TADDH	89.0	92.4	77.9	2.9	7.5	103.3
Baseline + FSFM + CGFM	90.0	93.5	79.1	3.0	7.7	102.8
Baseline + FSFM + TADDH	89.8	93.3	78.8	3.1	8.1	102.5
Baseline + CGFM + TADDH	90.2	93.7	79.3	3.0	7.9	102.8
Baseline + FSFM + CGFM + TADDH	91.0	94.6	80.2	3.2	8.5	102

## Data Availability

The data mentioned in this paper are available on request from the corresponding author.
